# The Importance of Psychosocial Factors in Aging: The Minority Aging Research Study

**DOI:** 10.1093/geroni/igab046.997

**Published:** 2021-12-17

**Authors:** Lisa Barnes, Brittney Lange-Maia, Carlos Mendes de Leon

## Abstract

Psychosocial factors can provide crucial insight into lived experiences that influence healthy aging. Though psychosocial factors are often used to explain health disparities seen between different racial/ethnic groups, within-group investigations can be particularly powerful for identifying culturally specific psychosocial factors that impact heterogeneity in aging among minority populations. The Minority Aging Research Study (MARS) is an ongoing, longitudinal epidemiologic cohort of 797 older African Americans from the Chicago, IL metropolitan area. Participants are on average 73.4 (standard deviation [SD]=6.6) years of age, 78.2% are women, and mean years of education is 14.8 (SD=3.7). At baseline, 75.3% of participants were without cognitive impairment, 20.8% had mild cognitive impairment, and 3.9% had mild dementia. Participants were recruited starting in 2004 and complete annual visits including a clinical evaluation, cognitive and motor testing, and assessment of risk factors related to Alzheimer’s Disease risk, including those hypothesized to be associated with a higher burden of cognitive impairment among older African Americans. This symposium will discuss the longitudinal association between John Henryism and cognitive function and decline (McSorley), participation in social activities and risk of all-cause mortality (Lamar), and the predictive relationship between experiences of everyday discrimination and incident disability (Lange-Maia). Finally, we will examine multilevel correlates—including environmental, sociocultural, behavioral, and biological factors—related to perceived stress (Glover). Mendes de Leon will critically consider what appear to be the most potent psychosocial factors for minority aging and possible implications of integrating these factors into interventions focused on promoting healthy aging among older African Americans.

